# Burnout and psychosocial working conditions in a multidisciplinary hospital radiology team: a cross-sectional study using the job demands–resources framework

**DOI:** 10.1186/s13244-026-02433-y

**Published:** 2026-10-15

**Authors:** Thomas Dertnig, Patrick Kutschar, Hans-Peter Wiesinger

**Affiliations:** 1 Institute of Diagnostic and Interventional Radiology and Neuroradiology, University Hospital Essen, Essen, Germany; 2 Paracelsus Medical Private University, Master Programme Public Health, Center for Public Health and Healthcare Research, Salzburg, Austria; 3 Institute of Nursing Science and Practice, Center for Public Health and Healthcare Research, Paracelsus Medical Private University, Salzburg, Austria; 4 Institute of General Practice, Family Medicine and Preventive Medicine, Center for Public Health and Healthcare Research, Paracelsus Medical Private University, Salzburg, Austria

**Keywords:** Absenteeism, Burnout, Job demands–resources model, Occupational health, Radiology

## Abstract

**Objectives:**

Burnout is a major concern in hospital radiology, but data on psychosocial work conditions remain limited. Using the job demands–resources (JD–R) framework, this study examined burnout and its associations with psychosocial work factors across professional groups in a German university hospital radiology department.

**Materials and methods:**

An exploratory cross-sectional survey was conducted among radiologists and non-physician staff. Burnout was measured using the Copenhagen Burnout Inventory (CBI); psychosocial work conditions were assessed with COPSOQ III. Descriptive analyses, correlations, and hierarchical linear regressions were performed to evaluate associations between job demands, job resources, and burnout dimensions (personal, work-related, and patient-related burnout).

**Results:**

Sixty-three participants (≤ 28 to ≥ 61 years; 37 women) were included. Personal and work-related burnout exceeded patient-related burnout. Non-physician staff reported higher personal burnout than radiologists. Job demands strongly correlated with personal (*r* = 0.686) and work-related burnout (*r* = 0.748), whereas associations with resources were weaker. In regression analyses, job demands were strongly associated with personal (*R*² = 0.62) and work-related burnout (*R*² = 0.60), without significant incremental resource effects. For patient-related burnout, job resources demonstrated an independent inverse association (total *R*² = 0.30). Higher burnout was associated with lower job satisfaction and greater sickness absence.

**Conclusion:**

In this exploratory single-centre study, burnout in hospital radiology was primarily associated with psychosocial job demands, particularly work–life conflict, workload, and role conflicts, whereas job resources showed weaker associations except for patient-related burnout. These findings suggest a predominantly demand-driven pattern within the JD–R framework and indicate that psychosocial working conditions may represent targets for intervention.

**Key Points:**

***Question*** Which psychosocial job demands and resources show the strongest associations with burnout among radiology staff in a university hospital department?

***Findings*** Work–life conflict, quantitative demands, and role conflicts showed the strongest associations with personal and work-related burnout among radiology staff.

***Critical relevance statement*** Burnout in hospital radiology was predominantly associated with organisational stressors, highlighting the relevance of psychosocial working conditions for organisational monitoring and intervention planning.

**Graphical Abstract:**

**Figure:**
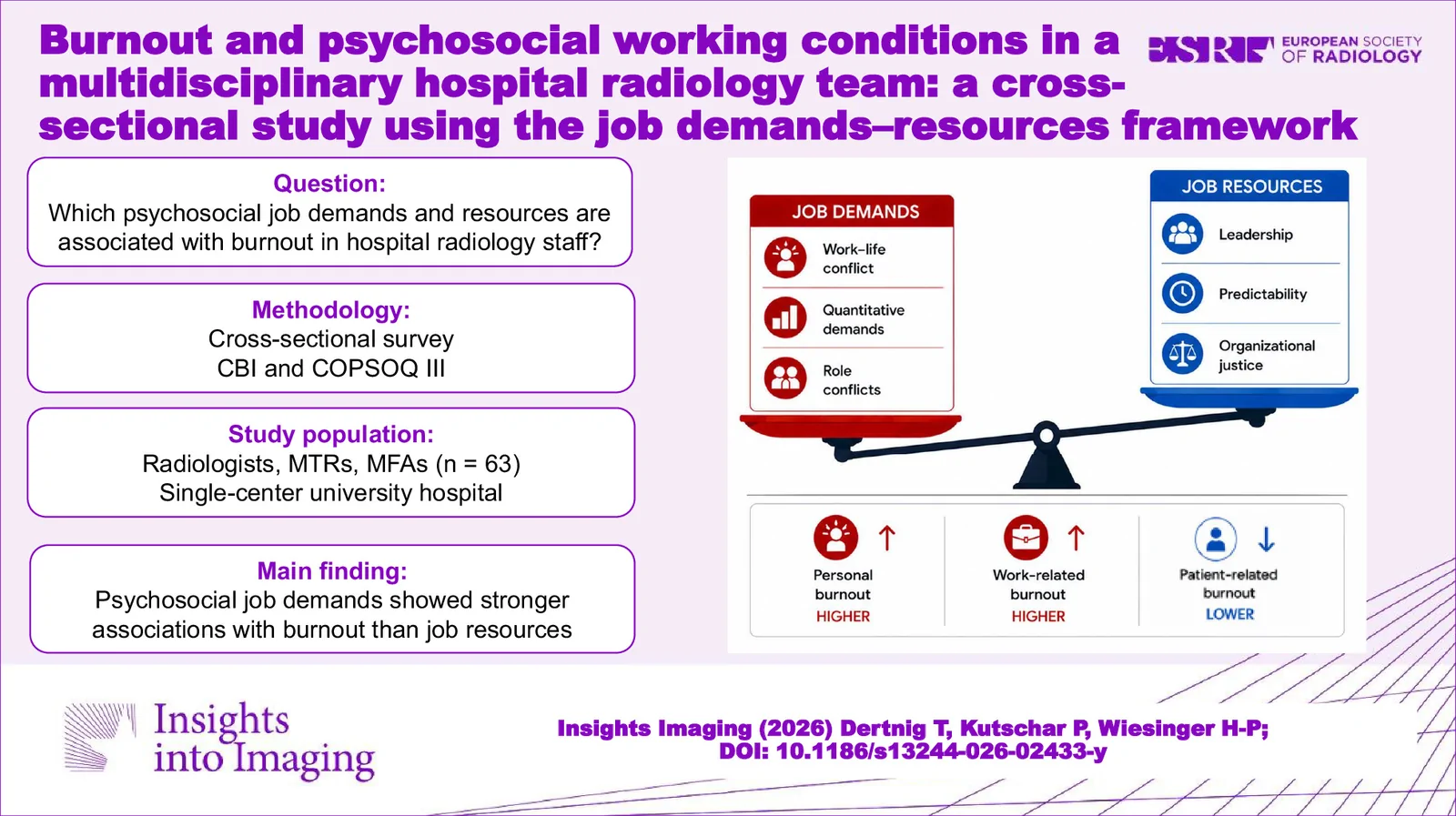

## Introduction

Burnout is a critical occupational health issue in healthcare [1], driven by chronic, unmanaged workplace stress and characterised by emotional exhaustion, depersonalisation , and reduced professional efficacy [2]. Radiology professionals are particularly affected [3–5], with surveys reporting burnout symptoms in 76.7% [6] of German radiologists and 71.9% of interventional radiologists in the UK [7]. Burnout is therefore not only a threat to individual well-being but also a determinant of diagnostic performance, patient safety, and workforce sustainability in radiology. It has been associated with reporting delays, reduced diagnostic accuracy, and increased risk of error, directly affecting clinical decision-making and patient outcomes. High diagnostic volumes, time-critical reporting, frequent off-hour coverage, and increasing administrative burden are associated with sustained cognitive and temporal demands and may contribute to work–life conflict [8–10]. These conditions also promote reporting delays and increased risk of diagnostic errors. Reduced face-to-face interaction and limited opportunities for informal peer exchange may further exacerbate professional strain [11].

Hospital radiology departments rely on multidisciplinary teams. Beyond physicians, medical-technical radiology assistants (MTR) and medical assistants (MFA) play essential roles in workflow, patient care, and operational continuity. These groups differ substantially in autonomy, decision-making latitude, and access to organisational resources, suggesting distinct vulnerability profiles [12–14].

The Job Demands–Resources (JD–R) model provides a framework for understanding how high job demands, if insufficiently balanced by resources such as control, support, or recovery opportunities, have been associated with burnout [14–16]. It proposes two complementary processes: a health-impairment pathway, in which excessive demands lead to exhaustion and burnout, and a motivational pathway, in which resources foster engagement and buffer the impact of high demands. This flexible framework allows occupation-specific demands and resources to be integrated and is therefore well suited for analysing heterogeneous, team-based environments such as hospital radiology departments, emphasising modifiable organisational factors rather than individual resilience.

Despite growing recognition of burnout in radiology, empirical data on psychosocial working conditions in European hospital radiology remain scarce, with most studies focusing exclusively on physicians. Little is known about how demand–resource patterns differ across professional groups within the same organisational context, and how these factors relate to burnout and work-related outcomes such as sickness absence in German hospitals.

Therefore, we aimed to (1) explore and quantify burnout and psychosocial working conditions across professional groups within a university hospital radiology department and explore role-specific differences, (2) examine associations between job demands, job resources, burnout, and sickness absence, and (3) assess the independent contributions of demands and resources to burnout within a JD–R framework. Burnout was the primary outcome, with psychosocial working conditions serving as explanatory variables. Identifying role-specific risk patterns may provide actionable targets for department-level interventions supporting workforce sustainability, maintaining diagnostic quality, and reducing reporting delays in hospital radiology.

## Material and methods

### Study design and setting

This cross-sectional observational study was conducted in the radiology department of the University Hospital Essen, Germany, between 01.12.2025 and 14.12.2025. Psychosocial working conditions, burnout symptoms, and sickness absence were assessed using validated instruments. Prior to the survey, a face-validity pretest was conducted with three radiology department employees from outside the study institution (excluded from the final sample) to assess comprehensibility, wording, and technical functionality of the online questionnaire. Based on their feedback, minor linguistic refinements were made. The institutional review board of the University of Duisburg–Essen reviewed the study protocol and waived the requirement for formal ethical approval (25-12602-BO). The clinic administration, institute leadership, staff council, and data protection officer were consulted, and no objections were raised.

### Participants and procedure

All professional groups working in the radiology department, including radiologists, MTRs, MFAs and registered nurses, were invited to participate. Participation was voluntary and anonymous. Data were collected via a secure web-based survey platform (LimeSurvey, LimeSurvey GmbH), accessible both during and outside working hours. Participants were invited via an initial email, followed by two reminder emails. Digital informed consent was obtained from all participants before survey completion.

### Instruments

Sociodemographic and occupational variables included age group, gender, professional role, years of experience, full-time vs part-time status, night/weekend duties, leadership responsibility, and self-rated job satisfaction. These variables were collected to characterise the sample and provide contextual information; detailed results are reported in the Supplementary Material. Sickness absence days were dichotomised (0–14 vs ≥ 15 days in the last 12 months) based on established public health thresholds to distinguish typical short-term illness from more substantial work-related health impairment. Sickness absence was not included in the primary regression analyses but was considered a contextual indicator of workforce capacity.

Burnout was assessed using the German version of the Copenhagen Burnout Inventory (CBI) [17]. The CBI conceptualises burnout as a state of prolonged physical and psychological exhaustion rather than a personality trait. The CBI distinguishes three analytically independent dimensions: personal burnout (six items), work-related burnout (seven items), and patient-related burnout (original CBI term: client-related burnout; six items). Personal burnout reflects general exhaustion irrespective of work. Work-related burnout captures exhaustion attributed to work demands. Patient-related burnout refers to exhaustion arising from interactions with patients or service recipients. Items were scored on five-point Likert-type scales and transformed to a 0–100 metric, with higher scores indicating greater burnout.

To operationalise the JD-R model, psychosocial working conditions were evaluated using the Copenhagen Psychosocial Questionnaire III (COPSOQ III) [18–20]. Responses to individual items were coded according to the COPSOQ III scoring manual and aggregated into validated scale scores, which were linearly transformed to a standardised 0–100 scale, with higher scores indicating higher levels of the respective psychosocial construct. The COPSOQ III instrument has been shown to reliably capture a broad range of psychosocial work factors, and its scales demonstrate satisfactory psychometric properties in large validation samples [20].

Reliability of the CBI in the present sample is consistent with prior reports in healthcare workers (Cronbach’s α = 0.82–0.86; Literature Benchmark 0.78–0.90) [21]. All COPSOQ III scales demonstrated acceptable to excellent reliability (α = 0.73–0.91; German validation α > 0.7) [20]. The vertical trust scale was the only exception, demonstrating very low internal consistency (α = 0.046) and was therefore excluded from further analyses. Because unreliable scales may introduce substantial measurement error and distort observed associations, Vertical Trust was not interpreted further. The low reliability may reflect heterogeneous interpretations of hierarchical trust and management structures across professional groups, as well as different supervisors within a large university hospital department.

Completion of the survey required approximately 20–30 min. Backward navigation between survey pages was disabled to minimise response revision. Participants were unable to save their responses and continue the survey later. Skipping individual questions was permitted, resulting in varying amounts of missing data across variables.

### Statistical analysis

Data were analysed using SPSS Statistics v29.0 (IBM Corporation). Independent-samples *t*-tests and Pearson correlations were conducted using listwise deletion for missing data, no imputation was performed. Descriptive statistics were calculated for sample characteristics, as well as burnout and psychosocial work condition scores. Group comparisons were restricted to radiologists and MTR due to insufficient sample size in other subgroups. Group differences were tested using independent-samples *t*-tests.

Pearson correlations were used to examine associations between psychosocial variables and burnout across the total sample. Effect sizes for Pearson correlations were interpreted according to conventional benchmarks (small: *r* ≈ 0.10, medium: *r* ≈ 0.30, large: *r* ≥ 0.50) [22].

To test the JD–R model, hierarchical linear regression analyses were conducted separately for personal, work-related, and patient-related burnout. Age group, gender, professional experience, night/weekend duties, employment status (part-time/full-time), and professional group were entered as control variables in Block 1, followed by aggregated job demands (Block 2) and aggregated job resources (Block 3). To reduce model complexity, aggregated scores were calculated as the unweighted mean of COPSOQ III scales for each participant. Because the number of COPSOQ subscales was high relative to the sample size, aggregated demand and resource scores were used to maintain statistical parsimony and avoid model overfitting. Job demands comprised quantitative demands, work pace, emotional demands, role conflicts, work–life conflict, and job insecurity; job resources comprised influence at work, possibilities for development, predictability, social support, quality of leadership, and organisational justice.

For inferential comparisons between professional groups, analyses were restricted to radiologists and MTR because MFA and other occupational groups were represented by only a small number of participants. Consequently, findings attributed to non-physician staff primarily reflect the MTR subgroup and should not be interpreted as representative of all non-physician radiology personnel.

Interaction terms between profession and psychosocial variables (mean-centred) were tested but were not significant (all *p* > 0.05); therefore, results are reported for the main-effects models. Regression models were not adjusted for additional sociodemographic or occupational variables given the limited sample size and the risk of model overfitting. The number of predictors was intentionally restricted to preserve model stability and reduce the risk of overfitting. Given the limited number of complete cases, coefficient estimates and *R*² values should be interpreted cautiously, as their stability and generalisability could not be assessed. Consequently, regression analyses should be interpreted as exploratory. Statistical significance was set at *p* < 0.05 (two-tailed).

## Results

### Participants

**Fig. 1:**
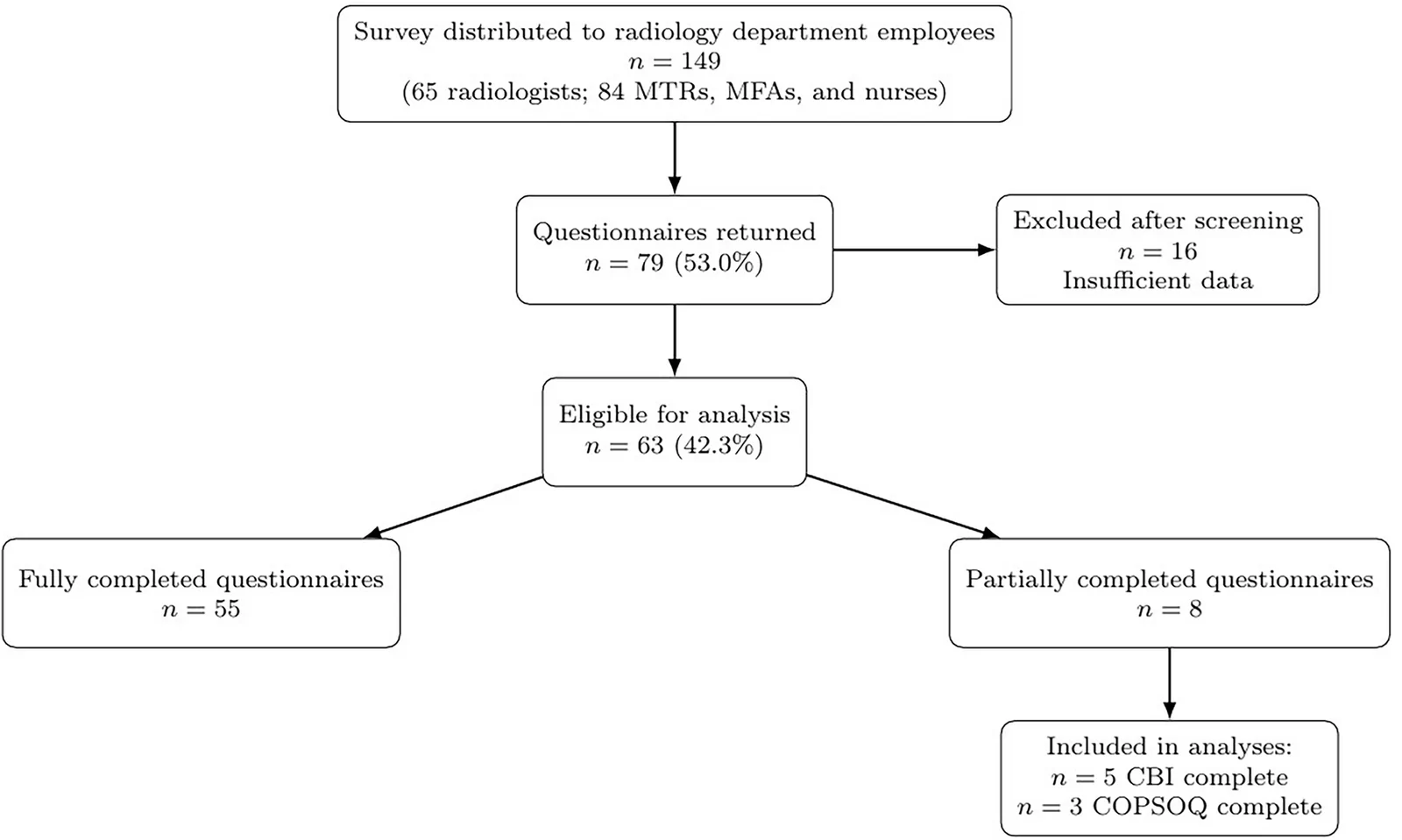
Flow diagram of participant inclusion. Of 149 employees, 79 responded (53.0%); 63 were included after exclusion of 16 incomplete responses (42.3%). Of these, 55 questionnaires were fully completed and 8 partially completed (CBI complete: *n* = 5; COPSOQ complete: *n* = 3)

The survey was distributed to 149 radiology department employees (65 radiologists and 84 MTRs, MFAs, and nurses). A total of 79 questionnaires were returned (response rate 53.0%). After excluding cases with insufficient data, 63 responses were included for analysis (net response rate 42.3%). Of these, 55 questionnaires were fully completed, while 8 were partially completed. Five partially completed questionnaires contained complete burnout (CBI) data, and 3 contained complete psychosocial working condition (COPSOQ) data; these were included in the respective analyses (Fig. 1).

The internal consistency of the CBI ranged from α = 0.82 to 0.86, and all COPSOQ III scales showed internal consistencies between α = 0.73 and 0.91, except for vertical trust (α = 0.046), which was excluded from further analyses. The final sample comprised radiologists, MTRs, MFAs, and registered nurses, with the majority working full-time and performing night or weekend duties (Table 1).

**Table 1:** Sociodemographic and occupational characteristics of the study sample (*N* = 63)

Variable	Category	*n*	%
Age (years)	*≤* 28	29	46.0
	29–44	20	31.7
	45–60	11	17.5
	*≥* 61	3	4.8
Gender	Female	37	58.7
	Male	23	36.5
	Diverse	1	1.6
	No answer	2	3.2
Professional role	MTR	32	50.8
	Radiologist	24	38.1
	MFA	5	7.9
	Other	2	3.2
Employment status	Full-time	49	77.8
	Part-time	14	22.2
Night/weekend duties	Yes	51	81.0
	No	12	19.0
Leadership role	Yes	12	19.0
	No	51	81.0
Experience (years)	*≤* 5	35	55.6
	6–15	14	22.2
	*>* 15	14	22.2
Work area	Diagnostic radiology	46	73.0
	Interventional radiology	16	25.4
Sickness absence (days)	0–14	51	83.6
	*≥* 15	10	16.4

*MTR* medical-technical radiology assistant, *MFA* medical assistant

“Other” refers to nursing staff (registered nurses) working within the radiology department

Percentages are based on valid responses per variable after excluding missing data

Other refers to nursing staff (registered nurses) working within the radiology department

### Burnout and psychosocial working conditions across professional roles

In the following, key findings are reported in the main text; full results, including non-significant findings, are provided in the Supplementary Material. Table 2 presents descriptive statistics for burnout dimensions and psychosocial working conditions stratified by professional group. Mean scores were higher for personal and work-related burnout than for patient-related burnout across the total sample and within both professional groups. Among job demands, the highest mean values were observed for work pace, emotional demands, and work–life conflict, whereas among job resources, the highest values were observed for meaning of work, sense of community, and social support, and the lowest for influence at work and job insecurity.

**Table 2:** Burnout scores and descriptive profile of COPSOQ III scales stratified by professional group

	Dept. (*n* = 63)	Radiol. (*n* = 23)	MTR (*n* = 30)	Mean diff.; 95% CI	*p*
CBI					
Personal burnout	48.8 ± 17.4 [0–83]	39.9 ± 17.6 [6–78]	54.2 ± 15.3 [22–83]	–14.4; –23.5 to –5.3	0.003
Work-related burnout	45.4 ± 15.5 [4–82]	40.4 ± 13.9 [10–71]	47.7 ± 16.4 [14–82]	–7.3; –15.9 to 1.3	0.094
Client-related burnout	29.0 ± 18.8 [0–83]	24.2 ± 16.7 [0–67]	29.3 ± 18.9 [0–83]	–5.1; –15.1 to 5.0	0.315

COPSOQ III scales			
	Dept. (*n* = 53)	Radiol.^a^	MTR^a^
Quantitative demands	53.9 ± 19.2	52.4 ± 15.6	57.1 ± 20.0
Work pace	75.9 ± 14.9	77.4 ± 15.6	75.9 ± 13.8
Emotional demands	52.7 ± 19.3	42.5 ± 16.0	60.5 ± 18.7
Role conflicts	50.0 ± 24.6	40.5 ± 22.7	59.7 ± 23.9
Work-life conflict	56.1 ± 29.0	57.1 ± 27.3	60.2 ± 29.6
Job insecurity	8.7 ± 21.0	15.5 ± 29.0	2.8 ± 6.3
Influence at work	42.0 ± 23.4	39.3 ± 25.7	43.5 ± 22.6
Possibilities for dev.	74.7 ± 21.7	80.6 ± 19.1	74.7 ± 20.9
Role clarity	76.9 ± 16.7	73.0 ± 15.6	78.7 ± 17.7
Quality of leadership	61.1 ± 27.4	55.4 ± 30.5	62.5 ± 25.5
Organizational justice	50.5 ± 23.4	48.8 ± 24.7	49.5 ± 21.8
Social support (coll.)	86.3 ± 18.1	89.3 ± 14.9	83.3 ± 19.6
Social support (superv.)	69.8 ± 28.3	67.9 ± 25.2	70.4 ± 30.3
Predictability	46.5 ± 25.4	40.5 ± 24.3	47.7 ± 25.5
Recognition	47.2 ± 29.7	46.6 ± 27.1	46.3 ± 30.9
Meaning of work	83.5 ± 17.6	82.1 ± 17.9	85.2 ± 17.3
Sense of community	85.4 ± 18.6	88.6 ± 14.9	82.4 ± 20.0
Insecurity	26.4 ± 30.0	23.9 ± 23.8	27.9 ± 34.1
Job satisfaction	71.2 ± 17.3	77.3 ± 18.8	66.2 ± 14.9
Self-rated health	56.6 ± 24.1	67.1 ± 28.2	48.4 ± 19.0

*CBI* Copenhagen Burnout Inventory, *COPSOQ III* Copenhagen psychosocial questionnaire III, *CI* confidence interval

All scales range from 0 to 100. Higher CBI values indicate higher burnout levels. Group differences are presented for CBI scales only. COPSOQ III scales are reported descriptively without inferential testing. All COPSOQ III scales with acceptable internal consistency are reported descriptively; the scale Vertical Trust (trust in management) was excluded due to insufficient reliability

^a^ The source population comprised 65 radiologists and 84 non-physician staff members. Sample sizes varied slightly across COPSOQ III scales due to missing data. For most scales, *n* = 22 for radiologists and *n* = 34 for MTR/MFA; for self-rated health, *n* = 22 and *n* = 32, respectively

Burnout scores varied considerably, with higher levels observed for personal and work-related burnout than for patient-related burnout (Table 2). MTR reported higher personal burnout than radiologists (*p* = 0.003), with no significant differences in the other burnout dimensions. Comparisons with other professional groups were not performed due to small subgroup sample sizes. Participants with ≥ 15 days of sickness absence showed higher levels of personal burnout (*t* = −2.39, *p* = 0.020) and work-related burnout (*t* = −2.03, *p* = 0.047) compared with those with fewer absence days. They also reported higher job demands (*t* = −2.08, *p* = 0.042). Descriptively, psychosocial working conditions were characterised by relatively high work pace and work–life conflict, alongside generally high levels of meaning of work and social support from colleagues.

### Associations between psychosocial factors and burnout

Personal and work-related burnout were most strongly associated with job demands, particularly work–life conflict (personal: *r* = 0.606; work-related: *r* = 0.703, both large effect sizes), followed by quantitative demands and role conflicts (moderate to large associations). Job resources were inversely associated with burnout, particularly organisational justice (*r* = −0.443 for personal and −0.455 for work-related burnout, both *p* < 0.001) and predictability (*r* = −0.324 for personal and −0.508 for work-related burnout). Patient-related burnout was positively associated with emotional demands (*r* = 0.298, *p* = 0.021) and role conflicts (*r* = 0.333, *p* = 0.011), and inversely associated with organisational justice (*r* = −0.348, *p* = 0.009) and predictability (*r* = −0.362, *p* = 0.005). Detailed correlations for all COPSOQ III subscales are provided in the Supplementary Material.

### Independent associations of job demands and resources with burnout

Job demands were associated with a substantial proportion of variance in personal (*R*^2^ = 0.62) and work-related burnout (*R*^2^ = 0.60) in Model 2 (both *p* < 0.001; Table 3). The addition of job resources in Model 3 did not significantly increase explained variance (Δ*R*² = 0.009, *p* = 0.322 for personal; Δ*R*² = 0.021, *p* = 0.126 for work-related burnout) and job resources were not independently associated with these outcomes. For patient-related burnout, job demands were significantly associated with burnout in Model 2 (*p* = 0.004), but this association was no longer significant after inclusion of job resources in Model 3 (*p* = 0.057). In contrast, job resources showed a significant independent inverse association in Model 3 (*p* = 0.044), with a total explained variance of *R*^2^ = .30. Hierarchical regression analyses were based on 52 complete cases after listwise deletion of missing values; therefore, the relatively high *R*² values should be interpreted cautiously as sample-specific estimates from exploratory models. Detailed analyses of individual demand and resource dimensions are reported in the Supplementary Tables.

**Table 3:** Hierarchical linear regression analyses examined associations between psychosocial working conditions and CBI burnout dimensions

	Model 1	Model 2			Model 3		
Predictor	*β*	β	95% CI	*p*	β	95% CI	*p*
(A) Personal burnout							
Job demands	–	**0.559**	0.50–1.10	< 0.001	**0.509**	0.40–1.10	< 0.001
Job resources	–	–	–	–	−0.116	−0.40 to 0.10	0.322
_* R*_^2^	0.374	0.619			0.628		
Δ*R*^2^	–	0.244		< 0.001	0.009		0.322
(B) Work-related burnout							
Job demands	–	**0.716**	0.70–1.20	< 0.001	**0.638**	0.50–1.10	< 0.001
Job resources	–	–	–	–	−0.182	−0.40 to 0.10	0.126
_* R*_^2^	0.202	0.603			0.624		
Δ*R*^2^	–	0.401		< 0.001	0.021		0.126
(C) Client-related burnout							
Job demands	–	**0.454**	0.20–1.10	0.004	0.313	−0.00 to 1.00	0.057
Job resources	–	–	–	–	−**0.331**	−0.70 to −0.00	0.044
_* R*_^2^	0.066	0.227			0.298		
Δ*R*^2^	–	0.161		0.004	0.070		0.044

Model 1 included age group, gender, professional experience, night/weekend duties, employment status, and professional group; Model 2 additionally included Job Demands (aggregated score); Model 3 additionally included Job Resources (aggregated score). Standardised coefficients (*β*), two-tailed *p*-values, *R*^2^, and ∆*R*^2^ are reported. Hierarchical regression analyses were based on 52 complete cases after listwise deletion of missing values

β = standardised regression coefficient; confidence intervals refer to unstandardised coefficients. Boldface indicates *p* < 0.05

## Discussion

This exploratory study identified substantial levels of burnout within a university hospital radiology department, with higher personal and work-related burnout than patient-related burnout. Job demands—particularly work–life conflict, quantitative demands, and role conflicts—showed the strongest associations with burnout, whereas associations with job resources were less consistent. MTR reported higher personal burnout than radiologists (*p* = 0.003), whereas differences in work-related and patient-related burnout did not reach statistical significance.

### Burnout and psychosocial working conditions across professional roles in hospital radiology

In this cross-sectional survey of staff in a German tertiary care radiology department, descriptive analyses indicated higher levels of personal and work-related burnout compared with patient-related burnout. The prevalence and pattern of burnout observed in this hospital radiology department are consistent with both national and international evidence. Recent German surveys have reported high levels of burnout among radiologists and identified workload, time pressure, and work–life interference as central correlates of exhaustion [6, 23]. International syntheses similarly demonstrate substantial heterogeneity in prevalence estimates depending on instruments and thresholds, but consistently highlight workload intensity, call burden, and work–life imbalance as main factors associated with burnout in hospital radiology [24–26].

MTR reported higher personal burnout than radiologists, while patient-related burnout showed smaller differences between professional groups; however, subgroup analyses should be interpreted cautiously given the modest sample size. This pattern is consistent with literature linking role stressors, emotional load, and system pressures to burnout risk [27]. Several organisational factors may contribute to this difference. Compared with physicians, MTRs often have less autonomy over scheduling and workflow organisation while simultaneously facing continuous patient throughput, operational interruptions, and time pressure. Opportunities to influence departmental processes and work organisation may also be more limited. Such differences may contribute to a greater imbalance between perceived job demands and available resources. However, because the present study was not designed to examine mechanisms underlying professional group differences, these interpretations remain speculative and hypothesis-generating. Across roles, personal and work-related burnout showed stronger associations with overall job demands, whereas patient-related burnout was less consistently related to these factors, suggesting a comparatively smaller contribution of patient-facing strain.

Burnout levels were lower than in some previous radiologist cohorts [4, 6, 7], although comparisons are limited by differences in instruments and the use of continuous rather than categorical burnout measures.

Among the assessed resources, quality of leadership showed moderate inverse correlations with personal and work-related burnout. Although leadership-related factors were incorporated into the aggregated job resource score used in the regression analyses, these findings are consistent with previous literature suggesting that leadership quality may influence employee well-being through communication, predictability, organisational support, and role clarity [28, 29]. Leadership-related factors may therefore remain relevant organisational targets despite the overall dominance of job demands in the present study. These observations are also consistent with contemporary occupational well-being frameworks [30], which emphasise the role of organisational culture, leadership, and workplace design alongside workload-related factors.

### Demand–resource correlates of burnout and sickness absence

Beyond burnout symptoms, sickness absence represents a pragmatic indicator of how psychosocial working conditions may translate into reduced staffing capacity and operational strain [10, 31]. Recent German primary-care data further demonstrate substantial and increasing durations of burnout-related sickness absence, underscoring its potential relevance for workforce capacity [32]. Employees reporting ≥ 15 sickness absence days showed higher personal and work-related burnout and higher job demands, while patient-related burnout and job resources did not differ, consistent with sickness absence reflecting accumulated occupational strain. Although directionality cannot be inferred from cross-sectional data, prospective evidence from the Danish PUMA cohort indicates that higher burnout predicts subsequent sickness absence, supporting the interpretation of absence as a downstream manifestation of sustained work-related burden [21].

Within the JD–R framework, the findings primarily support the health-impairment pathway, with job demands showing stronger and more consistent associations with burnout than job resources. In particular, work–life conflict, quantitative demands, and role conflicts emerged as the most relevant correlates, suggesting that sustained workload is a central driver of exhaustion. Job resources were inversely associated with burnout but added limited explanatory value when demands were high, consistent with longitudinal evidence supporting the JD–R model across healthcare settings [33]. Patient-related burnout showed a distinct pattern, with weaker associations with overall demands and relatively greater relevance of resources. This may reflect the structure of radiology work, where patient-related strain is less continuous but mediated through coordination and interaction with patients and clinical teams. In this context, organisational resources such as role clarity, predictability, and leadership quality may be particularly relevant [25, 31].

Night/weekend duties were associated with higher personal burnout and job demands, consistent with evidence linking increasing radiology on-call workload and shift work to fatigue and burnout [34].

### Clinical and organisational implications for hospital radiology departments

These findings are more consistent with organisational than individual correlates of burnout in this university hospital radiology department. Potential organisational measures may include reducing excessive workload, improving scheduling predictability, minimising role conflicts, and strengthening organisational resources such as role clarity and leadership quality [6, 24–27, 34]. Given the association between burnout, sickness absence, and workforce strain, burnout mitigation should be considered part of quality and capacity management in radiology departments.

Evidence consistently shows that burnout interventions are most effective when targeting organisational rather than individual factors [35]. In hospital radiology, the strongest correlates—work–life conflict and quantitative demands—are structural and reproducibly linked to burnout [6, 23, 36], which may support consideration of workload-adjusted staffing and predictable rostering. In addition, role conflicts and emotional demands, particularly among non-physician staff, represent relevant targets, with radiography research highlighting the contribution of unclear roles and sustained emotional labour under system pressure [27]. Strengthening organisational resources such as role clarity, predictability, organisational justice, and leadership quality may further mitigate burnout, especially patient-related exhaustion, as supported by meta-analytic evidence [37].

Integrating burnout surveillance with occupational health monitoring may be warranted, as reductions in burnout may be accompanied by lower sickness absence, consistent with prospective evidence linking burnout to future absence [21]. Beyond individual well-being, burnout is associated with organisational sustainability and quality-of-care outcomes, including disengagement and turnover. Two evidence-based principles can guide implementation: prioritising system-level stressor reduction and embedding monitoring into routine management cycles [35].

More broadly, the findings highlight the link between psychosocial working conditions and workforce sustainability. The co-occurrence of higher job demands, elevated burnout, and increased sickness absence indicates that adverse working conditions may be associated with reduced workforce capacity and operational strain. In the context of increasing imaging demand and workforce shortages, burnout should therefore be considered not only an occupational health outcome but also a system-level factor affecting service delivery.

### Limitations

Several limitations should be considered when interpreting these findings. Because of the cross-sectional design, the observed associations cannot establish temporal directionality between psychosocial working conditions and burnout. The study design further precludes causal inference and cannot disentangle whether high demands led to burnout or whether existing exhaustion increased perceptions of demands and work–life conflict. The study is also single-centre with a modest sample size and missing data across scales, which may reduce statistical power, inflate uncertainty around subgroup differences, and limit generalizability to other hospital radiology departments with different staffing models or case-mix. The survey did not assess person–organisation fit or alignment between individual and organisational values. Previous research suggests that perceived value incongruence may contribute to burnout and occupational distress among healthcare professionals [38]. Consequently, potentially relevant organisational influences beyond the JD–R dimensions assessed in this study could not be examined. The regression models were estimated in only 52 complete cases. Accordingly, the relatively high *R*² values may reflect sample-specific model fit and should not be interpreted as evidence of robust predictive performance. For regression analyses, individual COPSOQ dimensions were aggregated into overall job demand and job resource scores. Although this approach reduced model complexity, it may have obscured differential associations of individual psychosocial work factors with burnout dimensions.

An additional limitation is the exclusive use of self-reported measures. Burnout, psychosocial working conditions, job satisfaction, self-rated health, and sickness absence were assessed within the same survey, which may have increased the risk of common-method bias and inflated observed associations. Consequently, relationships between psychosocial work factors and burnout may partly reflect shared response tendencies rather than entirely independent constructs. The non-physician subgroup was dominated by MTR, while MFA and other occupational groups were only sparsely represented, limiting conclusions regarding non-physician radiology personnel as a whole. Future work should triangulate survey data with objective indicators (e.g., modality volumes, RVUs, turnaround times, call burden metrics, staffing ratios, and rota characteristics). Larger multicentre studies would enable more stable modelling, more detailed subgroup analyses, external validation, and prospective evaluation of temporal relationships. The findings should be interpreted as exploratory and hypothesis-generating and provide a basis for future multicentre investigations.

## Conclusion

This study demonstrates a substantial burden of burnout among staff in a hospital radiology department, with higher personal burnout among MTR compared with radiologists and elevated burnout and job demands among employees with night or weekend duties. Personal and work-related burnout were strongly associated with overall job demands, while job resources showed weaker associations except for patient-related burnout, which followed a distinct pattern. These findings suggest that burnout in this setting is more strongly associated with psychosocial job demands than with job resources. These findings require confirmation in larger multicentre cohorts and may not directly translate to smaller hospitals, outpatient radiology practices, or non-academic settings. Future studies are needed to determine whether organisational measures targeting workload, role clarity, and supportive work environments influence burnout levels.

## Supplementary information

Electronic Supplementary Material

## Data Availability

The data that support the findings of this study are available from the corresponding author upon reasonable request, subject to institutional data protection regulations.

## Abbreviations

CBI: Copenhagen Burnout Inventory
COPSOQ III: Copenhagen psychosocial questionnaire III
JD–R: Job demands–resources
MFA: Medical assistant
MTR: Medical-technical radiology assistant
