# Supplementary material for: Burnout and psychosocial working conditions in a multidisciplinary hospital radiology team: a cross-sectional study using the job demands–resources framework
Source: Insights Imaging. 2026 Oct 15;17:259. doi: 10.1186/s13244-026-02433-y (PMC13672748; doi:10.1186/s13244-026-02433-y)
Supplement: Supplementary file 1 [file 13244_2026_2433_MOESM1_ESM.pdf]

Burnout and Psychosocial Working Conditions in a Multidisciplinary Hospital Radiology Team: A Cross-Sectional Study Using the Job Demands–Resources Framework

Electronic Supplementary Material

Variable	Category	n	%
Job Satisfaction (n = 62)	Very satisfied	9	14.5
	Rather satisfied	20	32.3
	Neutral	25	40.3
	Rather dissatisfied	6	9.7
	Very dissatisfied	2	3.2
Sick Days (12 months) (n = 62)	0 days	9	14.5
	1–7 days	31	50.0
	8–14 days	11	17.7
	15–30 days	6	9.7
	>30 days	4	6.5
≥ 8 Sick Days	Yes	21	33.9
≥ 15 Sick Days	Yes	10	16.1
Extended Sick Leave ≥ 1 Week	Yes	21	33.3
	No	41	65.1
Reasons for Extended Sick Leave (n = 21; multiple answers)	Physical	18	85.7
	Psychological	8	38.1
	Family-related	1	4.8

Table S1. Job Satisfaction and Sick Leave. Job satisfaction responses range from very satisfied to very dissatisfied. Sick leave was assessed based on the number of days absent due to illness over the past 12 months, with additional thresholds (≥8 and ≥15 days, and ≥1 week) used to identify more substantial absences. Multiple reasons for extended sick leave were reported, including physical, psychological, and family-related causes. Percentages are calculated based on valid responses per item.

Scale	Mean	SD	Min	Max
**Job Demands**				
Quantitative Demands	53.9	19.2	8.3	83.3
Work Pace	75.9	14.9	50	100
Emotional Demands	52.7	19.3	8.3	91.7
Role Conflicts	50.0	24.6	0	100
Work–Life Conflict	56.1	29.0	0	100
Job Insecurity	8.7	21.0	0	100
**Job Resources**				
Influence at Work	42.0	23.4	0	100
Possibilities for Development	74.7	21.7	33.3	100
Role Clarity	76.9	16.7	33.3	100
Quality of Leadership	61.1	27.4	0	100
Organizational Justice	50.5	23.4	0	100
Social Support (Supervisor)	69.8	28.3	25	100
Social Support (Colleagues)	86.3	18.1	50	100
Sense of Community at Work	85.4	18.6	25	100
Predictability	46.5	25.4	0	100
Recognition	47.2	29.7	0	100
Meaning of Work	83.5	17.6	50	100
Self-Rated Health	56.6	24.1	0	100

Table S2. Descriptive Statistics for COPSOQ III Scales. All scores are scaled from 0 to 100, with higher scores indicating higher levels of the respective construct. Demands refer to stressors (e.g., workload, conflict), while resources represent protective factors (e.g., support, clarity).

COPSOQ III scale	Personal Burnout ρ (p)	Work-related Burnout ρ (p)	Client-related Burnout ρ (p)
Quantitative Demands	0.403 (p = 0.001)	0.394 (p = 0.002)	0.243 (p = 0.062)
Work Pace	0.270 (p = 0.035)	0.319 (p = 0.012)	0.165 (p = 0.214)
Emotional Demands	0.402 (p = 0.001)	0.378 (p = 0.003)	0.298 (p = 0.021)
Work–Life Conflict	0.606 (p < 0.001)	0.703 (p < 0.001)	0.288 (p = 0.028)
Role Conflicts	0.532 (p < 0.001)	0.461 (p < 0.001)	0.333 (p = 0.011)
Job Insecurity	0.175 (p = 0.176)	0.215 (p = 0.098)	0.084 (p = 0.531)
Influence at Work	−0.238 (p = 0.071)	−0.230 (p = 0.082)	−0.235 (p = 0.074)
Possibilities for Development	−0.180 (p = 0.174)	−0.209 (p = 0.113)	−0.241 (p = 0.066)
Role Clarity	−0.312 (p = 0.018)	−0.412 (p < 0.001)	−0.311 (p = 0.019)
Quality of Leadership	−0.369 (p = 0.004)	−0.359 (p = 0.006)	−0.246 (p = 0.064)
Organizational Justice	−0.443 (p < 0.001)	−0.455 (p < 0.001)	−0.348 (p = 0.009)
Social Support	−0.212 (p = 0.110)	−0.287 (p = 0.031)	−0.235 (p = 0.075)
Predictability	−0.324 (p = 0.012)	−0.508 (p < 0.001)	−0.362 (p = 0.005)
Meaning of Work	−0.260 (p = 0.051)	−0.319 (p = 0.014)	−0.221 (p = 0.097)

Table S3. Spearman rank correlations between psychosocial working conditions assessed with the Copenhagen Psychosocial Questionnaire (COPSOQ III) and burnout dimensions assessed with the Copenhagen Burnout Inventory (CBI). Correlation coefficients (ρ) with exact two-tailed p-values are reported. Positive coefficients indicate higher burnout with increasing job demands, whereas negative coefficients indicate protective associations of job resources. Sample sizes vary slightly due to missing data.

Target	Department-level actions	Priority subgroups	Evidence rationale
Reduce quantitative demands	Capacity planning aligned with exam volume and RVU burden; protected time for non-interpretive tasks; optimize protocols/triage; reduce “hidden work” and interruptions	Full-time staff; night/weekend staff	Imaging demand and on-call workload trends support workload as a core driver in radiology
Reduce work–life conflict	Predictable rosters; limit short-notice schedule changes; enforce recovery time after nights; clarify expectations about availability after hours	Night/weekend staff; full-time staff	Work–nonwork conflict is robustly linked to exhaustion/burnout across meta-analytic evidence
Reduce role conflicts	Define responsibilities and handoffs between radiologists, MTR, MFA; standardized escalation pathways; align performance expectations with realistic resources	MTR/MFA	Radiography evidence highlights system pressure and role strain as burnout-relevant; our subgroup findings point to role conflicts as salient
Address emotional demands	Structured debriefing for high-stress patient interactions; training for communication in acute settings; peer support for front-line staff	MTR/MFA; staff in high-acuity areas	Emotional labour is a recognized contributor to burnout in imaging teams; extended shifts amplify fatigue and strain
Strengthen role clarity (resource)	Update SOPs; onboarding refreshers; regular team briefings; role clarity checks during process changes	All staff; especially newer employees	Role clarity protective effects are consistent with occupational burnout literature and supported by our regression results
Strengthen organizational justice and leadership (resources)	Transparent workload distribution; participatory decision-making; leadership development; recognition and feedback loops	Whole department	Organizational strategies and participatory/workload interventions show measurable reductions in exhaustion in meta-analytic evidence

Table S4. JD-R-based organizational intervention targets for burnout reduction in hospital radiology departments. The table summarizes potential department-level interventions derived from the Job Demands–Resources framework, including target domains, priority staff groups, and supporting evidence from the current study and existing literature.
